# Understanding adherence to reactive treatment of asymptomatic malaria infections in The Gambia

**DOI:** 10.1038/s41598-021-81468-1

**Published:** 2021-01-18

**Authors:** Fatou Jaiteh, Joseph Okebe, Yoriko Masunaga, Umberto D’Alessandro, Jane Achan, Charlotte Gryseels, Daniel de Vries, Joan Muela Ribera, Koen Peeters Grietens

**Affiliations:** 1 Medical Research Council Unit the Gambia at the London School of Hygiene and Tropical Medicine, Fajara, The Gambia; 2 Medical Anthropology Unit, Institute of Tropical Medicine, Antwerp, Belgium; 3 Amsterdam Institute of Social Science Research, Amsterdam, The Netherlands; 4 Department of International Public Health, Liverpool School of Tropical Medicine, Liverpool, UK; 5 PASS Suisse, Neuchâtel, Switzerland; 6 School of Tropical Medicine and Global Health, Nagasaki University, Nagasaki, Japan

**Keywords:** Infectious diseases, Randomized controlled trials

## Abstract

The impact of different types of reactive case detection and/or treatment strategies for malaria elimination depends on high coverage and participants’ adherence. However, strategies to optimise adherence are limited, particularly for people with asymptomatic or no infections. As part of a cluster-randomized trial to evaluate the effect of reactive treatment in The Gambia, all residents in the compound of a diagnosed clinical malaria patient received dihydro-artemisinin–piperaquine (DP). Using a mixed method approach, we assessed which factors contribute to adherence among the contacts of malaria cases that showed no symptoms. Adherence was defined as the proportion of compound members that (1) returned all medicine bags empty and (2) self-reported (3-day) treatment completion. Among the 273 individuals from 14 compounds who received DP, 227 (83.1%) were available for and willing to participate in the survey; 85.3% (233/273) returned empty medicine bags and 91.6% (208/227) self-reported treatment completion. Although clinical malaria was not considered a major health problem, reported adherence was high. The drivers of adherence were the strong sense of responsibility towards protecting the individual, compound and the village. Adherence can be optimised through a transdisciplinary implementation research process of engaging communities to bridge the gap between research goals and social realities.

## Introduction

Malaria infected but asymptomatic carriers (i.e. apparently healthy individuals) can contribute to maintain malaria transmission when transmission intensity is low^1,2^. Unlike clinical malaria cases who may actively seek treatment, apparently healthy individuals do not seek treatment for malaria and can carry malaria parasites for relatively long periods^3,4^. These are targeted by several elimination strategies such as (1) mass drug administration (MDA), where treatment is provided to an entire population regardless of infection, and; (2) active case detection (ACD) where infections are detected actively by screening an entire population and then treating infected individuals^5^. Reactive case detection (RACD), an important sub-type of ACD, treats individuals living around a confirmed clinical case with or without screening^5,6^. The use of RACD is based on the clustering of malaria cases in space and time and the assumption that asymptomatic carriage is higher in the households of clinical malaria cases^6–8^. These approaches, however, face several constraints such as unstandardised procedures for screening and/or treatment, limited sensitivity of standard diagnostic tests to detect low-density infections, high implementation costs, exclusion of at-risk groups such as pregnant women and infants < 6 months old, potential population exhaustion, acceptability and adherence challenges, treatment tolerability, risk of drug resistance, and little efforts to contextualise these strategies^5,6,9^.

The success of these interventions depends heavily on treatment coverage (i.e. proportion of the target population that received the treatment) and adherence (i.e. proportion of the target population actually taking the treatment). A successful MDA campaigns is usually conceptualised as achieving at least 80% coverage and adherence of the target population^10–12^, which is often not reached. Moreover the barriers to treatment coverage have received more attention than facilitators that determine adherence to treatment of apparently healthy individuals^13,14^.

Integrating local, social contextual factors into the design of interventions, through community engagement and participation approaches, positively influences response to such interventions^9,11,15–20^. These approaches include adapting messages about the intervention to community concerns on the impact of the disease, transmission dynamics, treatment tolerability, local patterns of mobility, involvement of the community in the design and implementation of key activities and, actively responding to raised concerns and rumors^11,13,16,17^. These findings have been consistent in MDAs campaigns targeting neglected tropical diseases (NTDS)^21,22^. It is important to note that these observations were reported within the context of MDA with Directly Observed Treatment (DOT) performed by community volunteers, local health workers and/or external organizations^11,13,14,17,18,22^. DOT, as a drug delivery strategy, is recommended by the World Health Organization (WHO)—where feasible—to ensure adherence to treatment^12^. However, they are questions about the sustainability of this approach, its long-term feasibility, heavy burden placed on participants and ethical limitations. Studies on other infectious diseases have shown that adherence rates could be just as high when therapy is supervised by family members as when supervised by non-kin in a position of greater external control^23–25^.

This mixed-methods study was conducted within but independent of a cluster-randomised trial (CRT) evaluating a RACD-type intervention, consisting of the systematic self-administered treatment of compound members of a passively detected clinical malaria case with a 3-day course of dihydroartemisinin-piperaquine (DP) (Clinical trials.gov, NCT02878200, 25/08/2016) in The Gambia. The adult patient or caregiver, in case of sick minors, was expected to provide treatment to his/her not-tested compound members with the assistance of the village health worker (VHW). The complexity and effectiveness of this “self-administered” DP treatment was assessed and addressed through a transdisciplinary process of concurrent designing and implementing of an active community participation model^26^. This manuscript presents the factors contributing to treatment adherence of reactively identified at-risk compound members without symptoms.

## Methods

### Study site and population

The CRT and social science mixed-methods study were carried out in the North Bank Region of The Gambia. The main ethnic groups were Fula, Mandinka and Wolof, with the minority, belonging to the Bambara, Tilibonka and Turka*.* Mandinka and Fula are the main languages for communication and trade. The village structure comprises compounds defined by enclosed spaces containing one or several households belonging to the same extended patrilineal family. Gender ideology and roles situate men as household and compound heads. The compound head is often the oldest man in the family and his role includes overseeing family’s welfare, marriages, leading the agricultural productivity unit, household resource management and making health-related decisions. A typical compound has rooms for adult men, for married women which they share with their children, and a ‘boys house’ for circumcised boys. Farming is the main activity, with peanut as the main cash crop; and, rice, maize, beans and vegetables as subsistence crops. Most family income is supplemented by remittances from relatives living either in urban areas at the coast or abroad.

#### Malaria transmission

Malaria transmission is seasonal, occurring mostly between August and December. The main malaria parasite species is *Plasmodium falciparum*^27^. Malaria transmission has declined substantially over the last 20 years, with a parasite prevalence at peak transmission estimated at 5%^26^. This decline has been attributed to a scale up of many interventions such as universal coverage of bed nets, indoor residual spraying and improved case management^28^.

#### Access to medicine

Biomedical services consisted of a hospital in the main town and smaller health centers located along the main highway, providing basic care and health promotion. Road access is poor, and the common means of transport were horse- or donkey-drawn carts, walking and commercial vehicles. Villages with more than 400 residents have a VHW under the primary healthcare scheme^29^. VHWs are selected by community leaders and trained by the government to provide basic health services such as health education and case management of uncomplicated malaria; diagnosis with a rapid diagnostic test (RDT); and treatment with artemether–lumefantrine (AL). Malaria treatment is provided free of charge and, where necessary, VHWs refer patients to the nearest primary health facility. VHWs are supervised by community health nurses (CHNs).

#### Familiarity with MDA

The study population have been exposed to several biomedical interventions which also includes trials on MDA for malaria^30,31^. In 1999, MDA with artesuntate and pyrimethamine/suphadoxine (PSD) was carried out as part of a randomized double blinded study. Treatment was directly supervised by study nurses. Although the trial reported a coverage level of 85%, no overall benefit was observed. The study investigators indicated that perhaps a higher coverage was required for impact on malaria transmission^14,31^. A follow up qualitative study reported barriers to uptake as related to the lack of adequate information given to the community members regarding the timing of the dosing, the possible side effects of the drugs, and the indefinite roles for the delivery and assessment of treatment. The investigation called for the need for continued sensitization of community members to maintain and improve uptake of MDA^14^. A second MDA carried out between June and August 2014 involved one round of directly observed treatment with DP administered by trial nurses and fieldworkers^30^. A social science study ancillary to the trial reported that out of 3942 registered to participate, 67.9% adhered to the three daily consecutive doses^13^. The factors related to non-participation and adherence were long and short term mobility of the community members, perceived adverse drug reaction, rumours, logistical concerns and perceived lack of information^13^. Both studies highlight, the relevance of approaches which contextualize the intervention to the social context to maximise its potential for participation and treatment adherence^13^.

### Implementing reactive treatment in the trial

#### Implementation of research process

A transdisciplinary implementation research process identified social factors related to the effectiveness of the intervention and assessed solutions to potential problems identified by researchers, relevant community stakeholders, health service providers and policy makers. Potential changes and solutions were discussed in participatory workshops with community stakeholders, and implemented. The methodological approach, called the Community Lab of Ideas for Health (CLIH) consisted of (1) ethnography, (2) stakeholder analysis, (3) participatory workshops or ‘Labs’ and (4) monitoring and evaluation. This process led to the design of a medicine distribution strategy and messages for community sensitisation based on local social structures and social relations^26^.

#### Medicine distribution strategy

Once a clinical malaria case was diagnosed at a health facility by a study nurse, the adult patient or caregiver, in case of sick minors, received medicine bags with compound member’s name and dosing instructions to take home. The study nurse informed the VHW or village collaborator (VC)—a volunteer identified by the community in case of no resident VHW—of the event and compound where treatment has been sent. The VHW or VC then visited the compound the same day and distributed the medicines. When the clinical malaria case was diagnosed by the VHW, he visited the compound and distributed prepacked medicine to the compound members. VHW/VC distributed the medicines assisted by the compound/household head (or another family representative) with instructions on who should take the medicine, when and how. This included the specification that the 3-day course was to be taken once a day before breakfast for three consecutive days. Follow-up visits by the VHW/VC were scheduled for the day after treatment completion, at which time they checked whether the treatment had been taken, retrieved empty or unused medicine bags and inquired about any adverse events during the period. This information was relayed back to the study team, who recollected any unused pills and the medicine bags^26^.

### Study design

**Figure 1:**
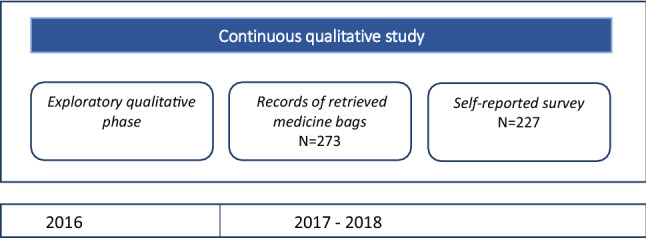
Flow chart of the study methodology and activities.

The study had a mixed-methods design, combining qualitative and quantitative research phases in all intervention villages (Fig. 1). The first phase of research (March and May 2016) involved gathering ethnographic qualitative data for an in-depth understanding of contextual factors influencing adherence to treatment. In the second phase (September 2017–January 2018), both quantitative and qualitative data were collected to assess adherence to treatment among those residing in the same compound with a diagnosed clinical malaria case. Quantitative data records of retrieved medicine bags and a self-reporting survey on adherence were complemented with qualitative semi-structured interviews to verify the findings from the prior quantitative and qualitative data.

### Qualitative study

#### Data collection

Field work was a continuous process following an emergent theory design where initial qualitative findings guided subsequent structured data collection. Participant observation involved daily-life observations and reiterated informal conversations. Continuous conversations including in-depth interviews with respondents helped in building confidence for discussions on treatment adherence.

#### Sampling

Purposive sampling was used throughout the study. The goal was to represent a variety of perspectives on the topic under study. The perspectives chosen for relevance of the study on the topic of adherence were from (1) general community members and (2) compound members who received DP treatment. Based on the principle of gradual selection, respondents were theoretically selected (based on emerging results) and categorised in relation to relevant criteria (age, gender, ethnicity, social status, occupation, previous malaria experience, treated with DP, etc.). In addition, snowball sampling techniques was utilized whereby respondents identified other potential respondents.

#### Data analysis

All interviews were recorded and transcribed for analysis. Research notes were taken during and after the interview process whenever it was informal or inappropriate to record. Data analysis was a flexible and iterative process where emerging findings and hypothesis were continuously tested in the field until saturation (i.e. no new findings emerging). The analytical induction process involved the iterative testing of theoretical ideas, which were used to refine and categorise themes grounded in the data whilst emerging themes were evaluated in dialogue with existing social science theory on adherence. This resulted in an adherence framework that was systematically applied in subsequent data collection and analysis. All interviews were systematised and analysed with NVivo 11 Qualitative analysis software (QSR International Pty Ltd. Cardigan UK).

### Quantitative study

#### Concept definition

For the quantitative study, “adherence” to DP was operationalised as the proportion of (1) compound members who returned complete medicine bags without pills *and* (2) compound members who self-reported to completing the 3-days treatment.

#### Data collection

Information on the medicine bags and pills was collected from the epidemiological records on all those who received the 3-day treatment. A structured, paper-based questionnaire survey evaluated the self-reported adherence of treated compound members. Questions assessed the use of DP, including the timing, frequency, dosage, observation and experienced adverse events. The forms were piloted before use to ensure clarity and to avoid translation errors in Fula, Mandinka and Wolof.

#### Sampling

All those who received the 3-day treatment were eligible for the survey. Children were represented by their designated caregivers. When targeted participants could not be reached, the reasons for non-participation were recorded in contact forms.

#### Data analysis

Completed questionnaires were double entered in Microsoft Access 15, verified and cleaned. Data was analysed using R (R version 3.3.0). Descriptive statistics were presented for the data and the association between related to adherence analysed using a regression model.

### Ethical approval

The study was approved by the Gambia Government/MRC Joint Ethics committee (SCC 1438v2, SCC 1484v2) and the Institutional Review Board of the Institute of Tropical Medicine, Antwerp, Belgium (1046/15, 1144/16). All methods were performed in accordance with the relevant guidelines and regulations. The interviewers followed the Code of Ethics of the American Anthropological Association (AAA)^32^. All interviewees were informed before the interview about the topic and types of questions and their right to decline participation, to interrupt or withdraw from the conversation. Informed consent was obtained from all participants and if participants are under 18, from a parent and/or legal guardian. Informed consent (oral) were preferred since the risk to the participant was minimal and the act of signing one’s name on a document could create mistrust since it is not customary practice within the local communities. Interviewees’ confidentiality was assured by assigning unique identifiers to the collected forms**.**

## Results

### Study participants

#### Qualitative study

A total of 108 in-depth interviews were carried out and 15 informal conversations were analysed across a range of respondents including village heads (Alkalos), index cases with clinical malaria and their caregivers, VHWs, compound and/or household heads and other community members (including those who received DP) (Table 1).

**Table 1:** Overview of respondents for in-depth interviews and informal conversations.

Adult participants	In-depth interviews and informal conversations
Village heads (Alkalos)	9
Alkalo’s family	6
Marabout (healer)	2
VHW (men)	5
Index cases with clinical malaria (men and women)	5
Index case caregiver (women)	8
Compound members who received DP (men and women)	11
Additional community members (men and women)	77
Total	123

#### Quantitative study

273 individuals were selected from the 14 compounds that received DP. 227 (83.1%) individuals were available and agreed to participate in the survey. The reasons for non-participation in the survey were moved away from the village (n = 21, 7.6%), travelling (n = 22, 8.0%), and inability to respond due to illness (n = 3, 1.0%). There were slightly more females (n = 120, 52.9%) than males (n = 104, 45.8%) and more than half of the individuals selected was single (n = 135, 59.4%). The biggest ethnic group was Fula (n = 150, 66.0%) (Table 2).

**Table 2:** Socio-demographic characteristics of respondents in survey (N = 227).

	Male (N = 104)	Female (N = 120)	NA
**Marital status**			17 (7.5%)
Single	75 (72.1%)	60 (50%)	
Married	20 (19.2)	47 (39.2%)	
Widowed/separated/divorced	0 (0%)	8 (6.7%)	
**Education level**			11 (4.8%)
Primary	23 (23.1%)	14 (11.7%)	
Secondary	8 (7.7%)	7 (5.8%)	
Tertiary	1(1.0%)	0 (0%)	
Arabic	23 (22.1%)	38 (31.7%)	
Others	44 (42.3%)	58 (48.3%)	
**Ethnicity**			
Mandinka	0 (0%)	0 (0%)	
Fula	68 (65.4%)	82 (68.3%)	
Wolof	13 (12.5%)	10 (8.3%)	
Bambara	6 (5.8%)	8 (6.7%)	
Turka	2 (1.9%)	3 (2.5%)	
Tilibonka	10 (9.6%)	14 (11.7%)	

### Quantifying treatment adherence to DP

#### Medicine bags and pill count

Treatment adherence to DP was estimated at 85.3% (233/273) when measured by the number of returned medicine bags and pills.

#### Survey

91.6% (208/227) of individuals reported to fully completing the treatment. Of those who participated, 8 individuals (3.5%) admitted to not completing the treatment; this was justified by bitter taste, experienced chest pain after intake, forgetfulness, prior meal intake, vomiting after intake, travelling and work pressure. About half (52.4%, 119/227) of the respondents reported that their treatment was observed; mainly by caregivers. The difference between men and women was not statistically significant. (Table 3). We see a difference in treatment observation by caregivers in different age groups. The proportion observed by caregivers is 91.5% (43/47) for 0–5 years, 89.8% (44/49) for 6–12 years, 64.3% (27/42) for 13–18 years and 8% (7/87) for adults (*p* value < 0.001).

**Table 3:** Self-reported adherence to DP treatment (N = 227).

	Male (N = 104)	Female (N = 120)	NA	*p* value
**Medicine use**			9 (4%)	0.33
Taken all pills	94 (90.4%)	114 (95.0%)		
Have pills remaining	4 (3.8%)	3 (2.5%)		
Pills received, not taken	1 (1.0)	0 (0%)		
Pills not received	0 (0%)	0 (0%)		
**Time of day of taking pills**			10 (4.4%)	0.12
Before breakfast	96 (92.3%)	117 (97.5%)		
After breakfast	2 (1.9)	0 (0%)		
**Number of pills taken per day**				
Once	96 (92.3%)	117 (97.5%)		
More than once	1 (1.0%)	0 (0%)		
**Number of days of taken pill**			13 (5.7)	0.36
1 day	1 (1.0%)	2 (1.7%)		
2 days	4 (3.8%)	1 (0.8%)		
3 days	88 (84.6%)	111 (92.5%)		
More than 3 days	2 (1.9%)	3 (2.5%)		
**Observation by**			19 (8.4%)	0.01
Caregiver	63 (60.6%)	56 (46.7%)		
Household head/compound head	1 (1%)	0 (0%)		
Village health worker	0 (0%)	0 (0%)		
MRCG nurse	0 (0%)	0 (0%)		
Not observed	30 (28.8%)	56 (46.7%)		

### Social drivers for adherence to DP

Social drivers for adherence to DP was researched using ethnographic methods. We grouped results into two themes to demonstrate (1) how malaria risk perception and treatment of healthy individuals are conceptualised; and (2) how social influence and responsibility reinforced adherence.

### Malaria risk perception

#### Perceptions on malaria transmission

Most people identified mosquitos to be the cause of malaria, during the rainy season. Increased malaria risk was associated with increased mosquito density related to presence of stagnant water (‘where the mosquitoes breed’) and ‘dirty areas’ with ‘lots of grass’ close to houses. Other perceived causes of malaria included ‘body heat transfer’ understood as the transmission of heat (and disease) through direct (e.g. sleeping together with a malaria case) or indirect (e.g. sleeping in the place where case was sleeping) contact, and eating from the ‘same bowl’, (as the disease could hide under the nails). These ideas shaped risk perceptions, which include staying close to an ill person and living too close to stagnant water. Furthermore, the idea that malaria could be hidden in the body without symptoms and remained transmissible was widely known and accepted. Indeed, most respondents reported that living in the same household/compound with a malaria case increased their personal risk of malaria infection. Most respondents perceived that clinical malaria was less of a problem now than before. The perceived decline was attributed to regular cleaning of the environment, spraying of houses with insecticides and access to and use of bed nets provided by the government and the presence of the Medical Research Council, The Gambia (MRCG) in the study area.

#### Perceived impact of malaria

The impact of malaria was explicitly linked to the loss of agricultural productivity and the economy of the compound. In the words of a respondent ‘malaria does not only affect the ill person, because when one gets sick one cannot work, and this affects the entire compound’ (Adult man, farmer). The compound constituted not only a residential space but also the production unit; therefore, protecting an individual from illness would avert its economic impact on the compound. An adult sick with malaria was considered to cost the compound about 3–5 working days and this had a significant impact on household economies (i.e. loss of 2–3 bags of harvest). In general, family members and friends often provided help for farming for free and out of solidarity. However, these gestures were limited during periods of heavy work pressure in the village. Few compounds with sufficient financial resources could offset some of this negative impact on productivity by securing a replacement worker during illness. The absence of key people on the farm could result in the loss of the crops due to the destruction by stray and wild animals.

Being sick could reduce my harvest. For example, when I start to dig the groundnuts and stop, the soil becomes dry so if I return back and start digging again all the groundnuts remain under the soil. This really affected my work which went backwards and is just yesterday that I was able to gather everything and bring it home (Adult man, farmer).

#### Conceptualisation of protection and prevention

Protecting oneself from acquiring illness or other mishaps was considered imperative. Commonly referred to as ‘*fankanta*’ (Mandinka), illness prevention is based on the idea that it is better to protect oneself before the illness (i.e. symptoms) comes out. Local self-protection practices ranged from wearing amulets or charms to prevent childhood illnesses, *jinn* (spirit) afflictions or ‘the evil eye’ during pregnancy. In this respect, informants considered the distribution of preventive medicine to the households to promote the health of its members as favourable. However, at the same time this conceptualisation had the potential to act as a barrier towards adherence to other malaria preventive measures. The study population had concerns on possible re-infection after treatment only if other infected members of their village remained untreated.

We believed that in taking the medicine we are protected from having malaria, but we also fear that if we live with the mosquitoes and they bite us we can still have malaria. Those compounds that don’t receive treatment are not protected. (Adult woman, farmer).

Informants explained that people who took the medicine were perceived as ‘protected’ from malaria for a period ranging from 6 months to 2 years, whilst those who did not were ‘not protected’.

### Social influence and responsibility towards DP intake

Individuals with authority in health-related topics and those well-known and respected in the villages were particularly influential in reinforcing adherence to DP.

#### Compound head

The compound/household heads were the leader of the therapy management group (TMG) (i.e. individuals who took charge of therapy management with or on behalf of the ill person)^33^. Compound heads are considered exemplary role models and trusted to take decisions on medical treatment for ill compound members. The compound head’s tasks included arranging for transport and cash for accessing health facilities, and to organize substitute workers on the farm. Where the compound head was away, the role passed to a brother or the first wife. Compound members frequently mentioned that family members would easily accept to take the medicine if the compound head accepted the distribution of medication within the compound. Compound heads often felt it was their responsibility to encourage and remind family members of adherence to treatment. They would show this by taking DP themselves. Although respect for the compound head was considered as a motivating factor, most adult members stated that ultimately their decision to accept and adhere to the treatment was based more on their need to protect themselves from malaria, maintaining good health and remaining productive within their agricultural work unit.

Younger children were particularly seen as vulnerable to malaria so their mothers or other caregivers regarded it as their responsibility to personally observe medication intake. Older children or teenagers were regarded as more difficult to supervise for treatment. They were often away from the compound since they participated in agricultural work or herding which required them to leave the compounds early and return late in the evenings. Teenagers often mentioned that seeing their parents taking the medicine motivated them to take it as well.

#### VHWs

At the micro-political level of the village, the VHW’s influence over people’s decision to take the medication was regarded as important. Community members considered the VHWs as belonging to their community and acting for the well-being of the residents. In addition, VHWs were seen as medicine providers due to their role in malaria case management, which was considered an important task. People’s trust in the VHW was reinforced when they felt better after the treatment he provided. The kinship relation of a compound with the VHW was considered an additional pressure for the compound to adhere to treatment. Some compound members regarded not taking the medication as ‘negative behaviour’ since it could make them look ‘bad’ in the eyes of the VHWs and other community members in the village. Finally, community members, including VHWs, viewed the VHWs collaboration with the implementing institution (MRCG) as positive as this improved their social status.

#### MRCG

Most informants reported overall trust in the MRCG as the implementing institution. They frequently expressed confidence in the efficacy of the DP treatment provided by the MRCG. Trust was often related to previous positive experiences with projects implemented by the institution, which reportedly reduced suffering from malaria and the related economic losses. Despite the positive responses towards the treatment provided, they also expressed concerns on the temporary solution to health service delivery in their villages which often deteriorated at the end of such projects.

## Discussion

With limited evidence-based approaches for optimising adherence to malaria elimination interventions, and the declining incidence in many pre-elimination settings, there is a growing need to understand drivers of treatment adherence in the apparently healthy individuals that may constitute the remaining parasite reservoir^34^. Adherence to DP was high when measured both by the number of returned medicine bags and pills (85.3%) as when self-reported (91.6%). Importantly, adherence was higher as comparison to a prior MDA trial implemented in the area with directly observed treatment (DOT)^13^. Within this study, adherence was reported as the proportion of the study population who completed the three doses of DP (67.9%) when directly observed by a team of nurses and fieldworkers^13^. This figure is far below the WHO minimum required adherence for successful MDA campaigns^11,12^. The challenges mentioned for the study population, included MDA related inconveniences such as the long waiting times, burden of procedures, timing and location^13^. Similar constraints were stated for another malaria MDA trial (which only reported coverage) implemented in the area^14^. The WHO acknowledges situations where DOT is not feasible, nonetheless recommendations for alternative strategies largely neglect the available resources of the family^12^. Our findings on the more complex reactive ‘’self-administered’’ treatment regime show that high treatment adherence can be achieved with the key involvement of family members or caregivers.

Treatment adherence was mainly reinforced by social factors. Despite the relatively low risk of transmission, motivation towards adherence was driven by a sense of responsibility to protect the individual, his/her family and the village, which was linked to the social structure and values of the community. Similar factors on treatment adherence during MDA have been described in the literature^13,21^. The perceived need of people adhering to preventive measures remained relevant as those living in the same compound with a malaria patient understood to be at risk due to both the presence of mosquitoes and an infected person. This in turn encouraged individual protection and responsibility towards preventing the impact of clinical malaria on the agricultural productivity of the compound^35,36^.

For preventive interventions, including those aiming at elimination, where families or larger units may be targeted, the individual may not have the complete autonomy to participate and adhere to treatment^37,38^. Often societal pressure against opting out is strong since the benefits of treatment can be perceived to go beyond the individual^38^. This research shows that the sense of pressure within the compound to comply with treatment was related to the perceived risk of further transmission to other compound members. Although rarely acknowledged, this clear benefit for adherence presents the ethical dilemma of putting non-compliers at risk of discriminatory social action and stigma^38^. Such adverse social impacts may result if interventions are implemented in ways which legitimize already held social prejudices against non-compliers^39^. This could manifest, for instance, if health messages relayed during community sensitization in elimination activities inadvertently label, those who do not adhere, as threats to its public health benefits^38,39^. In promoting adherence, approaches should go beyond encouraging individual responsibility^16,40,41^ and capitalize on shared motivations^9,42,43^. Meaningful solutions could be reached through a participatory process of dialogue bridging the relevance of scientific intervention goals with the social realities of the affected communities.

**Figure 2:**
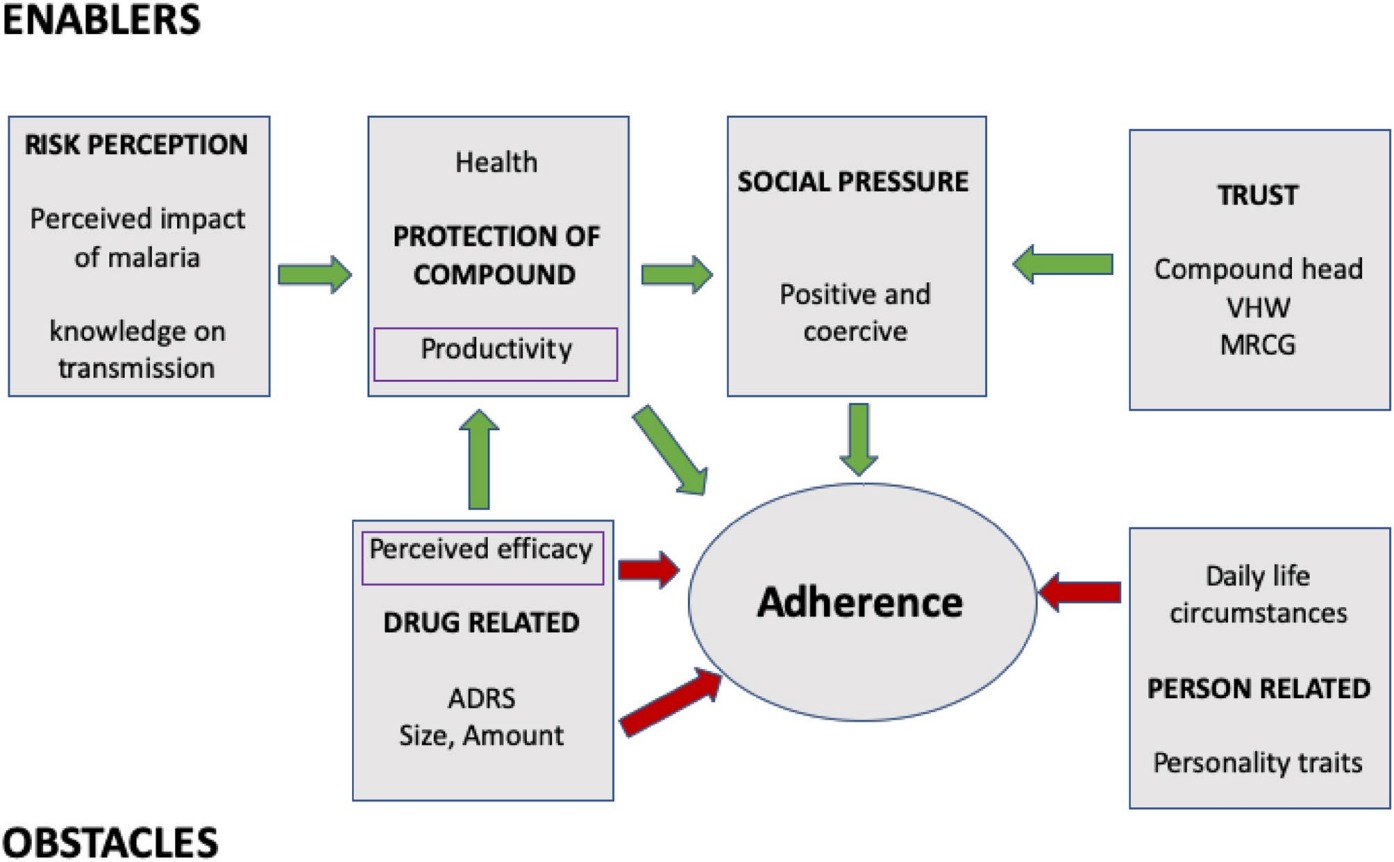
A general adherence model depicting the factors related to enablers and obstacles.

In discussing avenues for facilitating adherence to treatment, the important role of trust in the MRCG, in the specific Gambian context, cannot be understated. In the study setting, the communities had a long political and engagement history with the implementing institution wherein trust in their role was mainly based on their reputation for providing good health care against the backdrop of a limited health system^44,45^. VHWs who often collaborated with the MRCG in projects were considered as key stakeholders for further facilitating treatment acceptance and adherence^37,46^. Nonetheless, community’s trust in the VHWs and their socio-political leverage was fundamentally dependent on the VHWs perceived capacity to provide curative services for malaria. Community health providers may have insufficient status to motivate participation when their services are limited to health promotion rather than medicine provision^9,47^. Exploring and advocating for sustainable solutions supporting VHWs in the broader health system and beyond trials remains relevant^48,49^. While VHWs were perceived as key persons for biomedical health issues, the compound head was perceived as the gatekeeper of the compound. Involving compound heads in reinforcing adherence was crucial due to the local hierarchical structures and when linking adherence explicitly to the economic impact of the disease. Nevertheless, people often negotiate and have subtle ways of contesting authority, warranting the need for continuous dialogue to explore the supplementary role of other influential compound members such as caregivers and other household heads^50^ (Fig. 2).

Finally, high adherence to treatment was achieved within the context of a strategy of active community participation and collaboration between relevant community actors, health workers, researchers and trial implementers^26^. The reactive medicine distribution system developed through the transdisciplinary and iterative research process, shows that ethnographic methods can (1) provide contextualized relevant information; and (2) identify key community stakeholders (through stakeholder analysis) to facilitate discussions wherein researchers and community stakeholders negotiate the relevance of trial implementation. It further highlights the relevance of constant monitoring and evaluation of the implementation process to refine and improve developed strategies by utilising more of the community health resources.

## Conclusion

The transdisciplinary research process of implementing reactive treatment addresses the call for strategies to facilitate adherence of apparently healthy individuals to malaria elimination interventions. The factors which reinforced adherence were socially related, mainly driven by a sense of responsibility towards protecting the individual, compound and the village. Meaningful dialogue towards community engagement can help bridge the gap between intervention goals and the social realities of the community.

## Data Availability

For the qualitative study, the datasets generated and/or analysed are not publicly available due to the fact that participants did not consent to have their full transcripts made publicly available. However, the NVivo database with excerpts of the transcripts relevant to the study is available from the corresponding author on reasonable request. For the quantitative study, the datasets used and/or analysed are available from the corresponding author on reasonable request.
